# Host Immune Responses to Surface S-Layer Proteins (SLPs) of *Clostridioides difficile*

**DOI:** 10.3390/microorganisms11020380

**Published:** 2023-02-02

**Authors:** Harish Chandra, Rhett A. Kovall, Jagjit S. Yadav, Xingmin Sun

**Affiliations:** 1 Department of Environmental Microbiology, School of Earth and Environmental Sciences, Babasaheb Bhimrao Ambedkar University, Lucknow 226025, UP, India; 2 Department of Molecular and Cellular Biosciences, University of Cincinnati College of Medicine, Cincinnati, OH 45267, USA; 3 Department of Environmental and Public Health Sciences, University of Cincinnati College of Medicine, Cincinnati, OH 45267, USA; 4 Department of Molecular Medicine, Morsani College of Medicine, University of South Florida, Tampa, FL 33612, USA

**Keywords:** antibiotic-associated diarrhea, cell wall proteins, therapeutics, S-layer proteins, vaccine

## Abstract

*Clostridioides difficile*, a nosocomial pathogen, is an emerging gut pathobiont causing antibiotic-associated diarrhea. *C. difficile* infection involves gut colonization and disruption of the gut epithelial barrier, leading to the induction of inflammatory/immune responses. The expression of two major exotoxins, TcdA and TcdB is the major cause of *C. difficile* pathogenicity. Attachment of bacterial abundant cell wall proteins or surface S-layer proteins (SLPs) such as SlpA with host epithelial cells is critical for virulence. In addition to being toxins, these surface components have been shown to be highly immunogenic. Recent studies indicate that *C. difficile* SLPs play important roles in the adhesion of the bacteria to the intestinal epithelial cells, disruption of tight junctions, and modulation of the immune response of the host cells. These proteins might serve as new targets for vaccines and new therapeutic agents. This review summarizes our current understanding of the immunological role of SLPs in inducing host immunity and their use in the development of vaccines and novel therapeutics to combat *C. difficile* infection.

## 1. Introduction

*Clostridioides difficile*, a toxin-producing anaerobic bacterium, is an important opportunistic and nosocomial pathobiont in the gut that causes disease symptoms as a result of perturbations in the healthy microbiome due to a multitude of factors, including antibiotic use, genetic, exposome, microbial, and other host factors [1]. Selection and proliferation of *C*. *difficile* triggers the life-threatening condition of pseudomembranous colitis [2,3]. According to the latest estimates from the Centers for Disease Control and Prevention’s 2019 report, *C. difficile* caused 223,900 infections and 12,800 deaths in 2017 with a loss of $1 billion in the United States alone [4]. The treatment of the first episode of *C. difficile* infection (CDI) is achieved with antibiotics. However, the disease has a high level of recurrence—20–30% after the first treatment of an initial CDI and more than 50% after the first recurrence [5]. Therefore, urgent therapeutic intervention is needed to combat CDI worldwide.

One of the major molecular factors for *C. difficile* pathogenesis is the expression and secretion of two major toxins, TcdA and TcdB, encoded by the genes located within a 19.6-kb pathogenicity locus (PaLoc) in the *C. difficile* genome [6]. Regulation of toxin production and the various transcription factors involved in toxin production have been reviewed extensively by Chandra et al. elsewhere [6].

CDI pathogenesis starts with *C. difficile* spore ingestion/germination into vegetative cells, which germinate in the gut where they proliferate and colonize the intestinal mucosa [1,6]. The intestinal mucosal barrier (IMB) is the first line of innate defense against the pathobionts. The host IMB consists of various types of epithelial cells that are firmly joined with each other by tight junctions and covered with a thick protective mucus layer secreted by goblet cells [1]. Disruption of the IMB allows *C. difficile* to attach to the surface of the epithelial cells where elaboration of its virulence factors leads to the damage and manifestation of *C difficile* pathogenicity [1,6]. 

The bacterial cell wall in many Gram-positive and -negative species, including *C. difficile*, is associated with an abundant surface-exposed layer of protein molecules called surface-layer proteins (S-layer proteins or SLPs), predominantly made up of an abundant protein SlpA consisting of a low and high molecular weight domain, which are arranged as a paracrystalline regular two-dimensional array as seen by electron microscopy [7]. Other cell wall protein (Cwp) components of the SLP layer are less abundant and poorly characterized, but also play important roles in CD pathogenicity. In recent years, research on SLPs has gained increased attention, as these proteins have been shown to play key roles in surface adhesion, activation of Toll-like receptors, induction of cytokine production, and inflammasome activation as part of the host immune response besides their role in the growth and survival of the bacterium [7,8,9].

In this review, we discuss the host immune response to the major SlpA components and other less abundant Cwps in light of the recent knowledge of *C. difficile* SLPs and highlight their potential for use as a novel vaccine and therapeutic target highly relevant in CDI pathogenesis.

## 2. Host Innate Immune Responses against CDI 

It has been shown that non-toxigenic *C. difficile* (NTCD) strains upon colonization in animal models provide protection against the pathogenic strains of *C. difficile*. In the early 1980s, Wilson and Sheagren reported that hamsters colonized by an NTCD strain after having been sterilized with antibiotic cefoxitin were protected when challenged with a toxigenic *C. difficile* (TCD) strain [10]. However, treatment with other species, such as *C. perfringens*, *C. bifermentans*, *C. beijerincki*, *C. sporogenes*, and a heat-killed non-toxigenic *C. difficile* NTCD strain, failed to protect against CDI. Furthermore, the protection was lost when the colonizing NTCD was sterilized using vancomycin treatment before the challenge [11]. Encouraged by these findings, spores of the NTCD-M1 strain have been used in a limited number of clinical patients suffering from recurrent CDI (rCDI) with considerable success (about 50%) [12]. Currently, CDI treatment options are limited and heavily rely on the use of antibiotics, such as vancomycin, fidaxomicin, and metronidazole [13,14]. Excessive use of antibiotics leads to the dysbiosis of the healthy microbiome and further aids in the selection of pathobionts like *C. difficile* that can relapse later [1,15]. To effectively circumvent these challenges, alternate treatment methods need immediate attention. Some of these treatment options include neutralization of *C. difficile* toxins using monoclonal antibodies against TcdB, such as bezlotoxumab infusion, which prevents the toxin mediated damage of the gut epithelium [16]. Another fascinating method is restoration of healthy microbiome using fecal microbiota transplantation (FMT) from a healthy donor from among immediate family members. FMT has shown promising results against recurrent CDI with success rates up to 90% [17,18]. Unfortunately, these studies did not address the role of host immune response in protection of these patients. Therefore, it can be argued that live NTCD secretes some antigens/cell wall components that induce a strong immune response against the toxigenic TCD. Similarly, FMT protection is poorly defined. Therefore, understanding how FMT introduction induces host responses might help in the identification of key antigens, which in turn may help in a better understanding of the immune response and development of novel vaccines against CDI. 

In the gut, the host’s innate immune system is the first line of defense against an invading pathogen, which plays a crucial role in shaping and mounting a robust host adaptive immune response [1]. The innate response consists mainly of three parts: (i) intestinal epithelium and the mucosal layer (physical barrier), (ii) antimicrobial peptides, which are the excretory product of epithelial cells, Paneth cells, and some members of the gut microbiota (chemical barrier), and (iii) cellular responses by recruitment of innate immune cells such as neutrophils, eosinophils, macrophages, innate lymphoid cells (ILCs), and dendritic cells (DCs) that are orchestrated by multiple innate signaling pathways to combat the invading pathogen [1]. Host cells bear Pattern Recognition Receptors (PRRs), such as TLRs, on their surface that recognize certain conserved bacterial signatures on microbes called pathogen-associated molecular patterns (PAMPs). These PRRs are also known as Toll-like Receptors (TLRs). Upon recognition of these danger signals (PAMPs) by TLRs, the host cell triggers an immune response. In this regard, Toll-like Receptor 4 (TLR4) has been shown to recognize *C. difficile* danger signals, an action that participates in the initiation of the host inflammatory response. In this context, SLPs of *C. difficile* have been shown to interact with the host’s TLR-4 while *C*. *difficile* flagella interact through TLR5 [19,20].

## 3. S-Layer Proteins (SLPs) in *C. difficile*

In the recent decade, the surface layer (S-layer) proteins of *C. difficile* have received considerable attention. SLPs were first identified by Kawata et al. in 1984 and account for about 15% of the total cell mass [21,22]. SLPs are found in many diverse prokaryotic species. The majority of SLPs are arranged on the outermost surface of the cells as a single protein in a two-dimensional paracrystalline array [7]. In *C. difficile*, the S-layer consists mainly of the heterodimeric SlpA proteins. SlpA is a heterodimer consisting of a high molecular weight (HMW) protein and a low molecular weight (LMW) protein encoded by a single *slpA* gene; the LMW SLP forms the exposed upper layer while the HMW SLP forms the lower layer. The LMW SLP is unique in *C. difficile*. In the *C. difficile* 630 strain, the *slpA* locus is a 36.6 kb region that harbors 11 *slpA* paralogs. Furthermore, there are an additional 17 paralogs that are scattered throughout the genome [23,24]. These paralog genes are now named clostridial cell wall proteins (CwpX), where X denotes the paralogue number identified (X = 1–29) and are described in Table 1. However, four previously characterized Cwps known as SlpA, Cwp66, Cwp84, and CwpV were named before this new naming convention was adopted [23]. All Cwps are typical proteins that contain an N-terminal signal peptide and three putative cell wall binding domains with significant similarity to HMW SLP [25,26]. Different *C. difficile* strains have shown variation in the *slpA* locus and about 12 different S-layer cassette types have been documented. The other 28 Cwps act as accessory components, which are anchored in the polymerized paracrystalline layer, which accounts for ~5–20% of the S-layer [7].

## 4. Expression and Strain Variation of Cell Wall Proteins

In *C. difficile* strain 630, it has been reported that about nine Cwps encoding genes are expressed [25]. While *cwp2*, *cwp84*, *cwp6*, *cwp12*, *cwpV*, *cwp24,* and *cwp25* genes are expressed on the surface of the cell under normal growth conditions [44], *cwp66* and *cwp5* genes were expressed but were not found in cell surface extracts. In a separate study, Biazzo et al. analyzed *cwps* that are scattered throughout the genome of *C. difficile*. They observed that *cwp13*, *cwpV*, *cwp16*, *cwp18*, *cwp19*, *cwp20*, *cwp22*, *cwp24,* and *cwp25* genes are expressed and have well-conserved sequences, whereas *cwp17*, *cwp26*, *cwp27*, *cwp28*, and *cwp29* genes had significant variation in expression levels between ribotypes and were less conserved [45]. 

**Figure 1:**
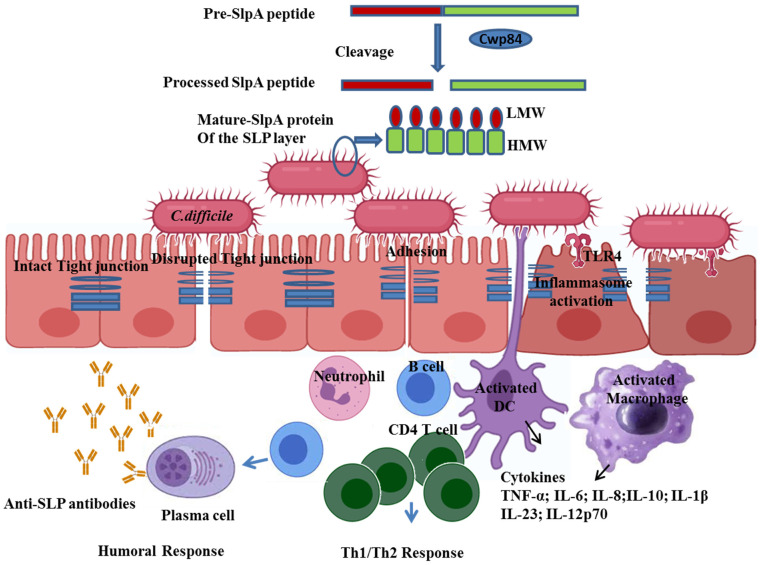
The SLPs of *C. difficile* mediate the adhesion and activation of the immune cells. Nascent SlpA peptide is cleaved by the protease Cwp84 into the LMW and HMW subunit, which forms the mature SlpA complex of the SLP layer of the cell wall [31,32,33]. SLPs mediate adhesion via TLR4 and disrupt the tight junction of the intestinal epithelial cells and further activate dendritic cells/macrophages, which in turn produce various cytokines and chemokines leading to the induction of Th1/Th2 and humoral response [9,27]. Interleukin (IL), Dendritic cells (DC), Low molecular weight (LMW), High molecular weight (HMW), Toll-like receptor 4 (TLR4).

Many *slpA* locus genes show significant variation between strains, particularly the surface exposed regions. For example, *slpA*, *cwp66*, *secA2*, and *cwp2*, have been shown to have high variation within the *slpA* locus (which forms a 10-kb cassette); so far 12 divergent variants of this cassette have been found as a result of homologous recombination between different genotypes [46]. According to Karjalainen *et al.*, *cwp66* shows only 33% identity between strains [26]. The *cwp2* variant has been replaced by a 23.8 kb predicted S-layer glycosylation gene cluster in the *slpA* locus [46]. SlpA is the most abundant SLP found in *C*. *difficile* cell surface extracts and is the main constituent of *C. difficile* SLP. The mature protein is cleaved after secretion into HMW and LMW protein forms by the action of a protease Cwp84 to form the heterodimeric complex H/L complex, which polymerizes to form the S-layer [47] (Figure 1). Inactivation of the *cwp84* gene in *C. difficile 630Δerm* resulted in an S-layer consisting of only an immature single chain SlpA with altered colony morphology, suggesting an important role of Cwp84 in the formation of the mature S-layer [32]. The presence of the *secA2* gene in the *slpA locus* is important for the transport of SlpA and other Cwps across the cytoplasmic membrane [48].

## 5. Functions of S-Layer Proteins

SLPs are involved in various functions in *C. difficile* biology (See Table 1), such as cell integrity, transport, forming of pores and anchors, degradation, host cell adhesion/invasion, immune system evasion, and protection from competing microorganisms [22]. Pechineet al. detected antibodies against the N-terminal and the C-terminal domains of the Cwp66 antigen in the sera of *Clostridium difficile*-associated disease (CDAD) patients [49]. In another study, Wright et al. separated the Cwps using 2D-PAGE and identified several Cwps (SlpA, Cwp2, Cwp5, Cwp84, Cwp18, Cwp19) that reacted with patients sera infected with *C. difficile* ribotype 017 strain suggesting the induction of strong immune response against SLPs [50]. Recently, Kirk et al. identified two *C. difficile* strains that lacked an S-layer and that were not susceptible to bacteriocin that forms pores and depolarizes the competing bacterial cells. However, these *C. difficile* strains showed significantly increased susceptibility to lysozyme and the antimicrobial peptide LL-37 and produced no disease symptoms of CDI in a hamster model of infection [51]. A recent study investigated the effects of SlpA isolated from three toxicogenic strains (RT126, RT001, RT084) on the expression of tight junction (TJ) proteins and induction of pro-inflammatory cytokines in the human colon carcinoma cell line HT-29. SlpA treatment significantly decreased the expression levels of the claudins family and JAM-A tight junction proteins (Figure 1) [9]. In addition, SlpA protein increased the expression levels of TLR-4 and induced the secretion of TNF-α, IL-1β and IL-8. These results demonstrate that SlpA protein mediated pathogenesis and induced inflammatory responses in the gut [9]. Therefore, it is reasonable to argue that SLPs are essential for *C. difficile* pathogenicity and immune responses. 

## 6. Immune Response to SLPs

Recent investigations have pointed out the important role of SlpA not only in bacterial survival and growth but also in shaping the immune response of the host. Immuno-proteomic-based approaches have shown the presence of anti-SLP antibodies in the sera of six patients infected with *C. difficile* ribotype 017, suggesting that SLP proteins are immunodominant and expressed during infection [50]. A study led by Bruxelle et al. showed an elevated level of anti-SlpA antibodies in CDI patients compared to healthy patients [52]. In another study, Negm et al. detected IgG antibodies in sera from a total of 327 individuals with CDI against SLP extracts from various *C. difficile* strains [53]. In addition to SlpA, the exposed C-terminal domain of the second most-abundant protein Cwp66 is highly variable while the N-terminal domain is well conserved. The variable C-terminal domains of Cwp66 and Cwp84 have been shown to be immunogenic in humans [49,54]. In addition, in CDI patients, the mean total anti-Cwp66 and anti-Cwp84 levels were lower than the healthy control group suggesting the protective nature of the antibodies. Therefore, SLPs, specifically SlpA, and other components have important roles in immune defense and are potential targets for immunotherapeutic and vaccine development as described in the following sections.

## 7. SLPs Mediate *C. difficile* Adhesion

*C. difficile* initiates infection by adhering to the intestinal epithelial cells leading to colonization. In this regard, bacterial SLPs such as SlpA and Cwp66 play a critical role in adhesion. It has been reported that variation in SLPs specifically SlpA in isolated *C. difficile* strains showed changes in adherence [55]. SLPs have been shown to bind different cell lines, such as human gastrointestinal cell lines of Hep-2 and Vero cells, and many proteins of the extracellular matrix. Further, treatment with anti-HMW-SLP antibodies inhibited *C. difficile* adherence. In addition, pre-treatment of the host cells with either purified SlpA subunits or anti-SlpA antibodies also prevented *C. difficile* adherence [55]. The largest member homolog of the SlpA family of proteins is the CwpV protein, which is expressed in a phase variable manner. In a separate study, it was shown that the C-terminal repetitive domain of the CwpV protein mediates *C. difficile* aggregation. Furthermore, this domain varies between strains and five antigenically distinct repeat types have been identified [40]. Another immunogenic protein named Cwp66 has been shown to have adhesion properties. Purified Cwp66 and antibodies against the N-terminal and C-terminal domains inhibited *C. difficile* adherence to cultured Vero cells suggesting the adhesion properties of Cwp66 [29].

## 8. Induction of Inflammatory Responses

Bianco et al. demonstrated the role of SLPs in the inflammation process. In that study, the SLPs from hypervirulent and epidemic (H/E) or non-H/E *C. difficile* strains were purified and studied in human monocytes and monocyte-derived dendritic cells (MDDCs) in terms of induction of immunomodulatory cytokines [interleukin (IL)-1β, IL-6 and IL-10] [56]. The study demonstrated that SLPs not only induced the maturation of MDDCs, with enhanced antigen-presenting activity but also induced the secretion of high levels of IL-10. However, no significant differences were found in the activation of monocytes and MDDCs by SLP preparations from H/E and non-H/E strains suggesting that SLPs do not contribute to the increased severity of the disease [56].

In another study, Ausiello et al. extracted SLPs from the clinical isolate C253 and showed that SLPs induced the secretion of enhanced levels of IL-1β and IL-6 pro-inflammatory cytokines in resting monocytes and induced maturation of human MDDCs, and enhanced proliferation of T cells [57]. Further, these treated MDDCs also released elevated amounts of IL-10 and IL-12p70 and induced a mixed Th1/Th2 immune response. TLR-4 played an important role in the SLP-mediated activation of DCs. It was demonstrated that SLPs could not activate DCs isolated from TLR4-mutant C3H/HeJ mice and failed to induce a subsequent Th immune response suggesting that SLPs activate innate and adaptive immunity mediated by the TLR4 receptor [19]. In another study, it was demonstrated in macrophages that SLPs from *C. difficile* induced a clearance response in terms of secretion of pro-inflammatory cytokines and chemokines with increased macrophage migration and phagocytotic activity [58]. Treatment with a p38 inhibitor reversed these responses suggesting the role of signaling molecules in SLP-mediated responses [58]. A very recent study reported inflammasome activation by *C. difficile* SLPs in a dose-dependent manner. Further, it was demonstrated that the cholesterol-rich microdomains (lipid rafts) on cell membranes helped in the binding of SLPs to the cell membrane. This was based on fluorescence microscopy, where it was shown that methyl-β-cyclodextrin (MβCD) treatment that depletes membrane cholesterol reduced SLP binding suggesting that SLPs recruit the lipid rafts, critical for *C. difficile* colonization and inflammasome activation [59].

These studies argue that *C. difficile* SLPs can activate the innate and adaptive immune responses, which are mediated partly by TLR4, suggesting an important role of SLPs in inducing an immune response. Therefore, these results also suggest the potential of SLPs as vaccine candidates against CDI.

## 9. Antibody Responses against CDI

Several immunological studies indicate that *C. difficile* infection and outcome depend on the intensity of the host immune response, a key factor in CDI pathogenicity. Thus, the inability to develop a robust antibody response may be a prognosis for the severity and recurrence of the disease [60]. In this regard, levels of antibodies against the major toxins have been correlated with the recurrence and severity of the disease [61,62]. Antibodies against the *C. difficile* surface components have been reported in the serum of CDI patients in earlier studies as well [63].

Drudy et al. evaluated humoral immune responses to *C. difficile* SLPs extracts in a cohort of 146 patients comprising 55 patients with *C. difficile*-associated diarrhea (CDAD), 34 patients with asymptomatic carriers, and 57 control subjects [64]. The study isolated high and low MW fractions extraction of the SLPs, which contained mainly the abundant protein SlpA. They measured serum antibodies using enzyme-linked immunosorbent assay (ELISA) in this cohort and found no significant differences in serum IgM, IgA, or IgG antibody levels. Interestingly, patients with recurrent episodes of CDAD had significantly lower IgM-anti-SLP levels than patients with single episodes. The study concluded that further studies should be done to determine specific anti-SLP antibody responses and protection studies using *C. difficile* SLPs [64].

Passive and active immunization using isolated extracts of HMW and LMW SLPs have shown encouraging results with enhanced survival rates in lethal hamster challenge models. O’Brien et al. demonstrated the protective response of anti-SLP antibodies on *C. difficile* infection in hamsters, where survival was significantly prolonged in the anti-SLP treated groups compared with control groups [65]. The protective effect of the antiserum was shown to be through the enhancement of *C. difficile* phagocytosis [65]. Eidhin et al., using active immunization, tested crude SLP extract containing equimolar amounts of the component LMW and HMW peptides of SlpA as a vaccine with different systemic and mucosal adjuvants in Golden Syrian hamsters and BALB/c mouse models. The study reported modest to poor antibody stimulation within different regimens and mouse models displayed stronger antibody responses to SLPs compared to hamsters [66]. In another study, Brun et al. examined the in vivo adjuvant activity of two peptides consisting of the receptor-binding domain of toxin A (TxA (C314)) and a fragment of SLP-36 kda from *C. difficile* strain C253 against fibronectin-binding protein A (FnbpA), a protective vaccine antigen against *Staphylococcus aureus* [67]. They evaluated the response using intranasal and subcutaneous routes and found that both fragments enhanced the production of circulating anti-FnbpA IgG and IgA. They concluded that these fragments when used as adjuvants differentially affect and polarize the immune system [67].

In another study, Shirvan et al. generated and expressed specific recombinant antibodies against SLPs such as Cwp66 and SlpA from *C. difficile 630* proteins using phage display and showed that these recombinant antibodies reacted to SLPs and their components in a strain-specific manner with high specificity [68].

Immune response and protection in a hamster model using the Cwp84 protease as antigen has been evaluated by several immunization routes [69]. The study found differential antibody titers based on the immunization routes. The best protection was observed via the rectal route of immunization. Further, immunized hamster groups resulted in a 26% weaker and slower *C. difficile* intestinal colonization after *C. difficile* challenge with a significantly higher survival rate (33% greater) than the non-immunized groups [69]. 

## 10. SLPs-Based Anti-*C. difficile* Therapeutics

Specific antibody-based therapeutics to neutralize *C. difficile* may be an effective strategy. Kandalaft et al. used single-domain antibodies to target SLPs [70]. The group prepared a panel of SLP-specific single-domain antibodies (VHHs) from the *C. difficile* hypervirulent strain QCD-32g58 (027 ribotype). Their results demonstrated a number of VHHs bound to QCD-32g58 epitopes located on the LMW subunit of SLP with high affinity. Further, they reported that these VHHs had binding specificity to the 001, 027 ribotypes, and a subset of these VHHs antibodies were broadly cross-reactive to the 012, 017, 023, and 078 ribotypes. These VHHs were also shown to inhibit the *C. difficile* QCD-32g58 motility in vitro [70].

The development of another precision antibacterial agent Av-CD291.2 by Kirk et al. has been reported that specifically kills *C. difficile* and prevents colonization in mice [51]. Av-CD291.2 has been shown to kill diverse *C. difficile* isolates based on the presence of SLP sequences in the *C. difficile* strains. The authors claim to have identified SLP null mutants containing a point mutation in the *slpA* gene, which are resistant to Av-CD291.2 agents. These mutants also had sporulation defects but were able to colonize the intestinal tract despite the attenuation of virulence in a hamster model [51]. Furthermore, they constructed a panel of Avidocin-CDs that kills various *C. difficile* strains in an SLP sequence-dependent manner suggesting an important role of these antibacterial based on SLPs to prevent CDI [51].

## 11. Concluding Remarks and Critical Unanswered Questions

The host develops a robust specific immune response against *C. difficile* toxins and surface components. SLPs have been shown to have a role in cell adhesion, induction of various cytokines through TLR4 activation, and activation of both the innate and humoral immune responses. However, studies on the activation of T-cell responses by SLPs are needed to further dissect the role of CD4 and CD8 cells. Based on the activation of the humoral response, these neutralizing antibodies against the toxins and the surface components can prevent clinical signs of CDI. Studies using active or passive immunization against SLPs have shown promising results, indicating that the strategy can be further developed into novel therapeutics against CDI pathogenicity. Much attention has been given to SlpA as a vaccine candidate; however, due to the high sequence variability of SlpA between strains, the vaccine may not be effective against all ribotypes. Therefore, epitope-based vaccines may be needed to circumvent this problem. In this regard, single-domain antibodies (VHHs) against SLPs are viable options, which bind the LMW-SLP subunit of the *C. difficile* with high specificity and have been shown to inhibit the motility of the *C. difficile* strains. Currently, understanding of the mechanistic role of SLPs and their paralogues in CDI pathogenesis, adhesion, and T-cell response is still at its initial stages and largely remains unexplored. Further studies are needed to dissect the molecular functions and specific immune responses of the SLPs, to help foster the rapid development of novel vaccine/drug targets and therapeutics to combat *C. difficile* infections.

## Figures and Tables

**Table 1:** Putative functions of the 29 *cwp* genes found in the *Clostridium difficile 630* genome.

Protein	Locus Tag	Molecular Functions	References
SlpA	CD630_27930	Mediate attachment to the cell surface through an interaction with PSII ‘PILL’ motif.	[27]
Cwp2	CD630_27910	Host cell adhesion.	[28]
Cwp66	CD630_27890	Adhesive properties. Stress tolerance and antibiotic resistance.	[29,30]
Cwp84	CD630_27870	Cleavage of SlpA to form HMW SLP and LMW SLP. Breaks down gelatine and the extracellular matrix proteins fibronectin, laminin, and vitronectin but not type IV collagen.	[31,32,33]
Cwp6 Cwp16 Cwp17	CD630_27840 CD630_10350 CD630_10360	Structurally similar to the RUNX family of eukaryotic transcription factors. Putative N-acetylmuramoyl-L-alanine amidase, autolysin	[34] [34] [34]
Cwp8	CD630_27990	Similar to Cwp2 with adhesive properties.	[28]
Cwp9	CD630_27980	Function not characterized. Similar to Cwp12 but shorter, lacks Big domain.	[35]
Cwp11	CD630_27950	Function not characterized. Similar to Cwp12, lacks Big domain.	[35]
Cwp12	CD630_27940	Presence of Bacterial immunoglobulin-like domains (Big domains) and CAP domains. Big domains are involved in host cell adhesion and invasion while CAP domains may have a role in signaling.	[35,36]
Cwp13	CD630_17510	Cleaves misfolded protein, ensuring a fully functional S-layer. Removes the pro-peptide from Cwp84.	[37]
Cwp14	CD630_27350	Presence of SH3 (Src Homology 3) domains with a hydrophobic ligand binding pocket, can bind with a PXXP motif.	[38,39]
CwpV	CD630_05140	Putative hemagglutinin/adhesion. Mediate cell aggregation and phage resistance, and act as a flagellar switch.	[40]
Cwp19	CD630_27670	Unknown function but may cleave peptidoglycan.	[41]
Cwp20	CD630_14690	Presence of lactamase domain. β-lactam antibiotics resistance, putative penicillin-binding protein.	[24]
Cwp21	CD630_31920	Has three PepSY domains. May be involved in protease inhibitors. Putative cell surface peptidase.	[35]
Cwp22	CD630_27130	Presence of YkuD domain, may be involved in peptidoglycan crosslinking. Cell wall biogenesis; peptidoglycan biosynthesis.	[42]
Cwp24	CD630_2193	Contain a C-terminal endo-β-N-acetylglucosaminidase domain, which may cleave peptidoglycan	[35,43]
Cwp26	CD630_12330	Contains one C-terminal PepSY domain that may be involved in protease inhibition.	[35]
Cwp5 Cwp7 Cwp10 Cwp18 Cwp23 Cwp25 Cwp27 Cwp28 Cwp29	CD630_27860 CD630_27820 CD630_27960 CD630_10470 CD630_18030 CD630_08440 CD630_04400 CD630_19870 CD630_25180	Cwps with unknown functions.	

## Data Availability

Not applicable.
